# Acrylamide in Food: From Maillard Reaction to Public Health Concern

**DOI:** 10.3390/toxics14020110

**Published:** 2026-01-23

**Authors:** Gréta Törős, Walaa Alibrahem, Nihad Kharrat Helu, Szintia Jevcsák, Aya Ferroudj, József Prokisch

**Affiliations:** 1 Institute of Animal Science, Biotechnology and Nature Conservation, Faculty of Agricultural and Food Sciences and Environmental Management, University of Debrecen, Böszörményi Street 138, 4032 Debrecen, Hungary; jprokisch@agr.unideb.hu; 2 Doctoral School of Animal Husbandry, University of Debrecen, Böszörményi Street 138, 4032 Debrecen, Hungary; ferroudj.aya@agr.unideb.hu; 3 Doctoral School of Health Sciences, University of Debrecen, Egyetem Tér 1, 4028 Debrecen, Hungary; walaaeb@mailbox.unideb.hu (W.A.); nihad.kharrat.helu@mailbox.unideb.hu (N.K.H.); 4 Institute of Food Technology, Faculty of Agricultural and Food Sciences and Environmental Management, University of Debrecen, Böszörményi Street 138, 4032 Debrecen, Hungary; jevcsak@agr.unideb.hu

**Keywords:** food toxics, heat-induced compounds, food processing, carcinogens, neurotoxicity, dietary exposure, risk mitigation, food safety, quality control

## Abstract

Acrylamide is a heat-induced food contaminant that can be formed through the Maillard reaction between reducing sugars and asparagine in carbohydrate-rich foods. It is recognized as having carcinogenic, neurotoxic, and reproductive risks, prompting global regulatory and research attention. This review synthesizes recent advances (2013–2025) in understanding acrylamide’s formation mechanisms, detection methods, mitigation strategies, and health implications. Analytical innovations such as LC–MS/MS have enabled detection at trace levels (≤10 µg/kg), supporting process optimization and compliance monitoring. Effective mitigation strategies combine cooking adjustments, ingredient reformulation, and novel technologies, including vacuum frying, ohmic heating, and predictive modeling, which can achieve up to a 70% reduction in certain food categories. Dietary polyphenols and fibers also hold promise, lowering acrylamide formation and bioavailability through carbonyl trapping and enhanced detoxification. However, significant gaps remain in bioavailability assessment, analysis of metabolic fate (glycidamide conversion), and standardized global monitoring. This review emphasizes that a sustainable reduction in dietary acrylamide requires a multidisciplinary framework integrating mechanistic modeling, green processing, regulatory oversight, and consumer education. Bridging science, industry, and policy is essential to ensure safer food systems and minimize long-term public health risks.

## 1. Introduction

Acrylamide is a small, highly reactive amide monomer that has been traditionally used in industrial applications, including in the synthesis of polyacrylamides utilized for water treatment, papermaking, and soil conditioning [1,2,3,4,5,6]. However, beyond its roles in manufacturing and environmental engineering, acrylamide is treated as an important public health concern because it forms as an unintended constituent in thermally processed foods. This issue is mainly associated with the formation of toxic chemicals via the Maillard reaction during high-temperature cooking, which has prompted new scrutiny by toxicologists, food scientists, and regulatory agencies [7].

Acrylamide’s toxicological profile is well-characterized. The compound is classified by the International Agency for Research on Cancer (IARC) as “probably carcinogenic to humans” (Group 2A) and has been associated with neurotoxic, genotoxic, and reproductive effects. These health concerns have spurred extensive research into its formation pathways, analytical detection, and effective mitigation strategies [8,9,10].

In 2002, the discovery of acrylamide in a wide range of common foods consumed regularly, like fried potatoes, baked goods, coffee, and cereals, revealed a new paradigm of dietary exposure to harmful compounds. Foodborne acrylamide, in contrast to industrially related exposure routes, can be formed via the Maillard reaction, a non-enzymatic browning process that accompanies high-temperature cooking; it predominantly involves the amino acid asparagine and reducing sugars [11,12,13,14,15]. However, acrylamide formation is not limited to the Maillard reaction. Scientific evidence indicates that additional pathways, including lipid oxidation, acrolein intermediates, and oxidative degradation of asparagine, also contribute to acrylamide generation in foods subjected to high-temperature and low-moisture conditions [16,17,18].

Our review critically summarizes the present status of knowledge on acrylamide in food systems from recent studies on its formation mechanisms and toxicological aspects as well. The food processing conditions, matrix composition, and chemical kinetics influencing acrylamide formation have been clearly described using both conventional and innovative analytical methods. International regulations, specifically EU Regulation 2017/2158 [19], concerning acrylamide limits, as well as novel approaches including enzymatic treatments, green processing of foods, and AI-powered monitoring, have also been summarized. The aim is to provide an integrated and interdisciplinary overview of acrylamide and inform the development of manufacturing technologies that make food safer.

## 2. Methodology

A comprehensive literature review was conducted using major scientific databases, including ScienceDirect, SpringerLink, PubMed, and Google Scholar. Relevant studies were identified through a systematic combination of keywords, such as “acrylamide,” “Maillard reaction,” “thermally generated contaminants,” “detection methods,” “food toxicology,” and “mitigation strategies.” The search covered peer-reviewed original studies and review papers published in English between 2019 and 2025, with the selective inclusion of earlier seminal works to provide historical and mechanistic context.

To ensure the inclusion of the most recent advancements, particular emphasis was placed on studies published after 2020, including recent exposure assessments, analytical innovations, and patented mitigation technologies. Only full-text publications with transparent methodologies were eligible. Exclusion criteria encompassed abstracts, inaccessible articles, and studies with insufficient methodological clarity. Following a two-step screening of titles/abstracts and full texts, articles were assessed for topical relevance, methodological rigor, journal impact indicators, and author credentials. Extracted data on acrylamide formation, toxicological effects, detection techniques, and mitigation strategies were synthesized thematically. The resulting evidence was structured into comparative tables and graphical summaries to highlight emerging research trends and knowledge gaps in food safety and processing.

## 3. Acrylamide: Definition and Properties

Acrylamide is a colorless, odorless organic compound belonging to the alkylating agent group of chemicals. It can be prepared from acrylic acid in mild conditions and is produced naturally during the thermal treatment of foodstuffs containing high levels of carbohydrates [20]. This Maillard reaction includes the interaction of reducing sugars and amino acids at high temperatures, leading to browning and the generation of a number of potentially harmful compounds, of which acrylamide is one example [21].

Familiar food sources are French fries, roasted coffee, potato crisps, canned olives, breakfast cereals, and a variety of baked goods. Small amounts can also be found in raw vegetables such as carrots, corn, and potatoes [22]. Chemical attributes are summarized in Table 1.

**Table 1:** Chemical properties of acrylamide (Prop-2-enamide).

Property	Description
Molecular Formula	C_3_H_5_NO
Other name	Prop-2-enamide
Molecular Weight	71.08 g/mol
Melting Point	84.5 °C
Boiling Point	125 °C (under reduced pressure, e.g., 25 mmHg) or 192.6 °C (at atmospheric pressure)
Water Solubility	Very high (204 g/L 25 °C-on)
Toxicity Concerns	Neurotoxic, carcinogenic, genotoxic
Reactive Characteristics	Vinyl-amide compound with alkylating properties

Sources: [23,24].

For acrylamide exposure through drinking water, the Joint FAO/WHO Expert Committee on Food Additives has set an acceptable daily intake of 0.1 mg/kg body weight [25]. Nevertheless, other dietary sources contribute much more significantly to total exposure, with a much lower safety margin of 2.7-fold compared with 125-fold via water exposure. In most countries, no regulatory actions limiting acrylamide exposure through food have been taken as yet, despite the call for urgent action to reduce health risks [26,27,28].

## 4. Formation Mechanism of Acrylamide

Acrylamide is a polar, water-soluble organic compound used in many industries, from food processing to beverages. It can play multiple roles in food systems, including as a bleaching agent, a gelation aid, and a component of polymer-based adsorbents [29,30]. Nevertheless, acrylamide is also inadvertently formed during food processing, primarily through conventional high-temperature methods such as frying, baking, roasting, and drying [31].

Research has been conducted on this compound (since 2002) to explore its mechanisms of formation and potential strategies to reduce the risks. However, data on long-term exposure and associated health risks remain limited. Continued investigation and strengthened regulatory oversight are necessary. Foods commonly exceeding 100 ng/g acrylamide formed through the MR include fried and baked goods, coffee, and toasted cereals [21,32,33].

Different biochemical pathways can lead to the formation of acrylamide. For instance, the nucleophilic reaction of asparagine with reducing sugars results in a product that undergoes further dehydration to form acrylamide. Also, asparagine can be hydrolyzed, and its product undergoes dehydration to yield acrylic acid as an intermediate [34]. Cooking at high temperatures induces lipid oxidation, forming ROS that facilitate acrylamide formation through intermediates such as acrolein [35]. It should be mentioned that asparagine-derived intermediates may also react with dicarbonyl compounds like hydroxymethylfurfural (HMF), which makes a huge contribution to acrylamide levels [36].

**Figure 1:**
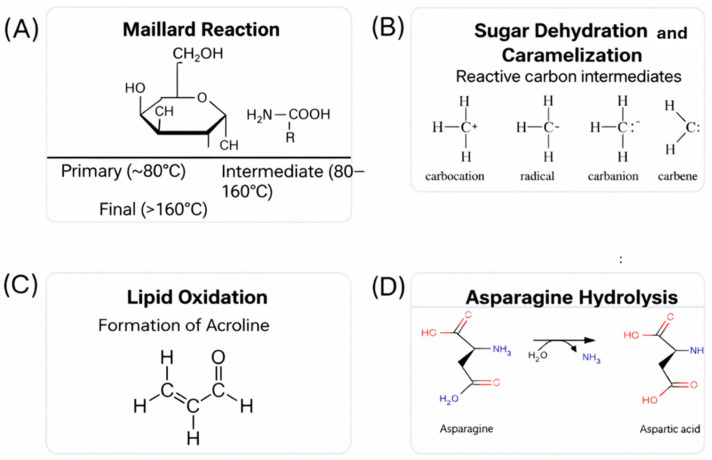
Formation pathways of acrylamide in foods: (**A**) Maillard reaction, (**B**) sugar dehydration/caramelization, (**C**) lipid oxidation, (**D**) asparagine hydrolysis.

Figure 1 presents the most important biochemical mechanisms, such as the Maillard reaction (A), the dehydration of sugar (B), lipid oxidation (C), and hydrolysis of asparagine (D), each leading to the generation of acrylamide under thermal conditions. The above-mentioned pathways revealed the complicated and multifactorial nature of acrylamide formation [21,35].

**Figure 2:**
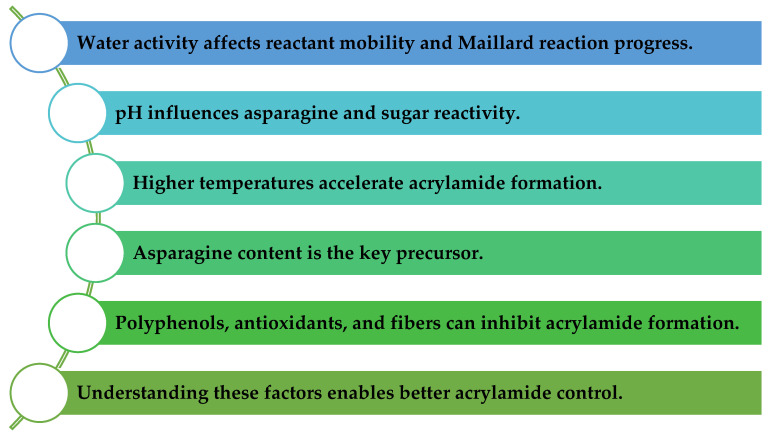
Key factors influencing acrylamide formation and control in food matrices.

Among the most effective control measures are pH adjustment, enzymatic mitigation strategies (for instance, asparaginase), and manipulation of the food matrix composition, as is visible in Figure 2 [37,38].

## 5. The Presence of Acrylamide in Different Food Systems

Overall, recent studies highlight that acrylamide and other neo-formed contaminants (NFCs) are prevalent in high-temperature-processed foods, such as potato-based snacks, coffee, and cocoa [39,40,41,42]. In Belgium, acrylamide was frequently detected in potato products, vegetable crisps, coffee substitutes, and cereals, with levels significantly lower in oven-baked products compared to deep-fried ones [39]. In Bosnia and Herzegovina, some local potato snacks and biscuits exceeded benchmark acrylamide levels, indicating gaps in mitigation practices [41]. Cocoa processing, particularly bean roasting and wet conching, can increase acrylamide and furan levels, although final chocolate products generally show low concentrations [40]. Given the widespread consumption of these foods, reliable analytical methods are essential to monitor NFCs and guide strategies to minimize dietary exposure [42].

As shown in Table 2, the highest acrylamide levels are consistently reported in low-moisture-, high-temperature-processed foods such as potato crisps, biscuits, and instant coffee. In contrast, raw or boiled foods generally contain negligible amounts. This distribution highlights the dominant role of processing conditions over raw material origin in determining dietary acrylamide exposure, underscoring the need for product-specific mitigation strategies rather than universal approaches.

## 6. Health Implications of Acrylamide

### 6.1. Carcinogenic Potential

Acrylamide was first identified as a food processing byproduct in 2002 and has since become a health concern due to its classification as a probable human carcinogen, Group 2A, by the IARC [21]. Animal studies provide abundant evidence for its carcinogenicity, with acrylamide inducing tumors in multiple rodent organs [33]. Human epidemiological studies are generally considered inconclusive, showing inconsistent associations between dietary acrylamide and cancer risk [51].

The EFSA, the JECFA, and the U.S. EPA have recommended continuing to monitor acrylamide exposure, with a special focus on children [52]. Based on their findings, the researchers promoted the establishment of indicative limits on acrylamide content in foods in several regions, which was accompanied by the proposal of mitigation strategies in the form of ingredient selection, formulation modification, and optimization of thermal processing parameters [53]. Regardless, more intensive follow-up studies will be needed to understand the human-specific mechanisms of acrylamide-induced tumorigenesis and to explain discrepancies between humans and animal models [54]. Table 3 presents animal studies on the carcinogenic, toxicological, and biochemical effects of acrylamide in different species and through different routes of exposure.

### 6.2. Neurotoxicity

Acrylamide is an established neurotoxin in both humans and animals. Experimental studies have shown that it causes neuropathy through various mechanisms, including axonal degeneration, oxidative stress, and disruption of neurotransmission. Repeated exposure to acrylamide in laboratory animals results in sensorimotor impairment, whereas occupational exposure in humans has been linked to peripheral neuropathy [63]. It forms covalent bonds with neuronal proteins, leading to disruption of function [64]. Neurotoxic effects indicate the dire need for strict safety assessments and exposure controls, especially for exposure through food [65].

Table 4 summarizes the major neurotoxic effects of acrylamide evidenced in both in vitro and in vivo models. As illustrated in the table, several neuronal and glial cell lines, such as PC12, SH-SY5Y, U251, BV-2, and human embryonic stem cells, showed dose- and time-dependent oxidative stress, excitotoxicity, apoptosis, and disturbed neuronal differentiation following acrylamide treatment.

### 6.3. Reproductive Effects

Exposure to acrylamide and glycidamide impairs oocyte quality, meiotic progression, and cytoskeletal integrity and enhances DNA damage, apoptosis, and autophagy. All these changes point toward compromised fertility and embryonic development, as reflected by the lower rates of blastocyst formation and litter sizes in animal models [74,75]. In males, both chemicals decrease sperm count, motility, and viability and increase sperm malformations. They induce DNA damage in sperm, disturb steroidogenesis, and alter antioxidant defenses in Leydig and Sertoli cells, all contributing to reduced fertility with possible transgenerational effects [76,77]. Glycidamide, formed through CYP2E1-mediated metabolism of acrylamide, exhibits particularly strong genotoxicity, resulting in DNA adducts and heritable mutations in germ cells. Epigenetic modifications and altered gene expression were also observed in reproductive tissues [76,78]. International benchmark levels and regulatory guidance for acrylamide in selected high-risk food categories are overviewed in Table 5.

## 7. Regulatory Guidelines and Recommendations

### 7.1. Global Regulations

The European Commission, Codex Alimentarius, the U.S. FDA, and JECFA have established various approaches to minimize acrylamide exposure in foods, ranging from binding EU regulations and voluntary Codes of Practice to guidance-based strategies and scientific risk assessments. These frameworks emphasize good manufacturing practices, process control, raw material quality, continuous mitigation, and the application of the Margin of Exposure (MOE) and ALARA principles. Key differences and strategies among these authorities are summarized in Table 6.

National and international agencies recommend establishing product-specific monitoring programs while considering variability in food composition and processing conditions [54]. Member states are also encouraged to keep reference laboratories, adopt standardized methods of sampling, and include proper labeling to inform consumers about acrylamide levels in foods prepared by heat processing [86]. All the above steps will ensure that there is fair trade taking place and, on the other hand, help minimize health risks related to trade. Notably, industrially prepared foods have low amounts of acrylamide compared to those prepared at home or within catering settings, which points to the role of controlled manufacturing practices [52].

**Figure 3:**
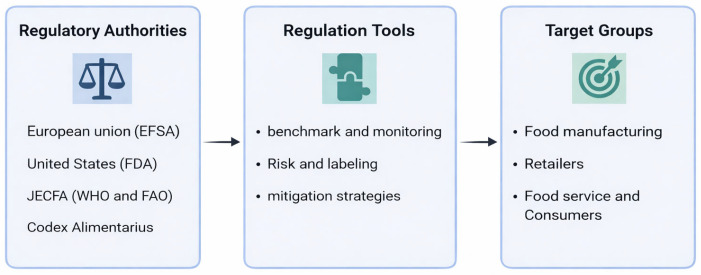
Regulatory tools, stakeholders, and benefits of acrylamide control in the food industry.

Figure 3 provides a global view of a regulatory framework for acrylamide in foods, showing major authorities, regulatory tools, and target groups.

Although mitigation efforts have achieved measurable reductions, a significant proportion of products still exceed the European Union benchmark values; this suggests that initial progress has reached a plateau. This emphasizes the need for sustained and pragmatic improvements following the ALARA principle of As Low As Reasonably Achievable and foreshadows the probable future adjustment of regulatory thresholds. One important development is that the color parameter a* is recognized as a robust proxy for acrylamide concentration, so product appearance is, in effect, linked with compliance status, and the parameter offers food manufacturers a possible metric for real-time control and process optimization [87]. The public health and industry outcomes expected from complying with mitigation strategies are reduced consumer exposure, improved food safety, harmonized international standardization, transparent and informed consumer choices, industry innovation and accountability, better monitoring and risk management, and public health benefits.

### 7.2. Food Safety Standards

With long-term exposure and potential carcinogenic properties, dietary acrylamide is considered a public health concern by the European Food Safety Authority (EFSA). In 2015, dietary exposure was quantified by EFSA, particularly within potato-based and cereal-based foods, suggesting average intakes of 0.4–0.57 µg/kg body weight/day [88]. Subsequently, mitigation benchmarks and best practice guidelines were provided within Regulation (EU) 2017/2158 to reduce AA levels in foods when processed above 120 °C, as occurs with frying, baking, and roasting. Food categories included potato snacks, bakery goods, and coffee. Manufacturers will not be found liable if they demonstrate compliance with these controls. Ongoing risk assessments and the industry’s adaptation of mitigation strategies, such as ingredient selection and cooking processes, remain key to reducing public exposure. Acrylamide detection is widespread in common foods such as potatoes, coffee, mushrooms, and cereals. Quantification generally occurs through Gas Chromatography–Mass Spectrometry (GC–MS) and High-Performance Liquid Chromatography (HPLC) approaches, though refinement of extraction and detection methodologies continues to be developed due to matrix complexity and differences across laboratories. Analytical results form the basis of regulatory benchmark levels, precautionary principles, and other mitigation strategies to manage the public’s long-term dietary exposure [89].

To enhance the objectivity and comparability of acrylamide risk assessment results, future studies should incorporate Margin of Exposure (MOE) calculations, defined as the ratio between a toxicological reference point (e.g., BMDL10) and estimated human dietary intake. The MOE approach is widely recommended for acrylamide because it combines lower confidence limits for benchmark doses with human exposure data, and both JECFA and EFSA use MOE values to assess health concerns. Furthermore, an MOE of 10,000 or greater (based on BMDL10) is generally considered to indicate a low public health concern for genotoxic carcinogens, highlighting the usefulness of MOE for harmonized risk characterization [46,88].

## 8. Methods for Reducing Acrylamide in Food

### 8.1. Cooking Techniques and Ingredient Modifications

Minimizing acrylamide formation in foods is not a single-task activity; rather, it involves multiple integrated approaches based on how acrylamide forms and which ingredients enhance its development. A major influencing factor relates to the manner of food preparation [79,83]. Some simple yet effective changes in cooking can lead to reduced levels of acrylamide. For example, adjusting the time of cooking and temperature alone can drastically reduce acrylamide levels. Soaking raw potatoes in water before frying helps in washing away the sugars that lead to the formation of acrylamide [19,84]. Similarly, proper storage is also crucial; keeping potatoes cool but not refrigerated prevents the accumulation of sugars, thereby reducing acrylamide formation during cooking [85]. Drying and pasteurization techniques allow better management of heat exposure, thus resulting in safer final products. Ultimately, the acrylamide content is dependent on a combination of environmental factors, the food item composition itself, and how it has been processed, particularly in potato-based products [23]. Another important approach to acrylamide reduction involves making appropriate changes to the ingredients themselves. For example, adjusting the pH with the use of salts such as sodium, calcium, or potassium can help limit acrylamide content, especially in cereal-based foods [85]. Similarly, adding components such as whey protein, modified starch, or glycine alters the dough chemistry, thus reducing acrylamide content without compromising taste and texture [86]. Other ways include replacing reducing sugars with their non-reducing counterparts, such as sucrose, which can lower acrylamide risks in snack products. Preheating ingredients prior to frying or extruding them has been found to be useful, maintaining quality while reducing acrylamide levels [87]. Furthermore, the type of sweetener utilized has a significant bearing on the degree of browning and acrylamide generation; thus, sweeteners like maltitol and glucose syrups need to be judiciously selected to achieve an optimal balance between sensory properties and food safety [88]. Many recent studies have further supported the empirical rationale behind these cooking-related mitigation strategies. Table 7 presents a chronological summary of findings from selected research conducted in the period between 2013 and 2024 on how changing cooking techniques and applying pretreatments have influenced acrylamide formation in fried starchy foods.

### 8.2. To What Extent Can Antioxidants Affect the Acrylamide Content?

Emerging studies indicate that dietary antioxidants and prebiotic fibers may also modulate acrylamide absorption and metabolism within the gastrointestinal tract [30,31]. The polyphenol-rich matrices have been shown to reduce acrylamide bioavailability, including extracts from mushrooms and green coffee beans, most likely due to binding or neutralization of reactive carbonyl intermediates formed during digestion [70,94].

Recent studies have demonstrated the potential of food polyphenols to inhibit acrylamide formation in food matrices and reduce its in vivo bioavailability. In starch-based baked systems, the addition of green tea polyphenols resulted in reductions in acrylamide levels as high as 48%. In the presence of soluble dietary fibers, these reductions increased to as much as 64%, indicating a synergistic interaction between polyphenolic compounds and matrix components [34].

In model bread formulations, the addition of individual polyphenols, for instance, catechin and ferulic acid, can result in reductions ranging from 16% to 95%, where ferulic acid especially has a significant inhibitory effect [35]. A recent study related to grape-derived polyphenol extracts showed a 60% reduction in acrylamide content in potato chip production. However, it should be mentioned that these compounds were ineffective under simulated physiological conditions, so their mitigation potential is considered to be highly dependent on the type of food [36]. Polyphenol-rich insoluble dietary fibers have demonstrated functional stability, while also maintaining their carbonyl-trapping capacity after in vitro digestion, resulting in physiological relevance for diminishing acrylamide absorption in the gastrointestinal tract [37].

In a controlled human clinical trial of bamboo leaf polyphenols, a significant reduction in internal acrylamide exposure was reported. This decrease can occur due to enhanced urinary excretion of acrylamide metabolites, which may be due to the active detoxification processes mediated by polyphenols in vivo [38], so dietary polyphenols have many roles to play in the mitigation of acrylamide, both during food processing and after food intake. Further studies are needed on the interactions with acrylamide exposure.

**Figure 4:**
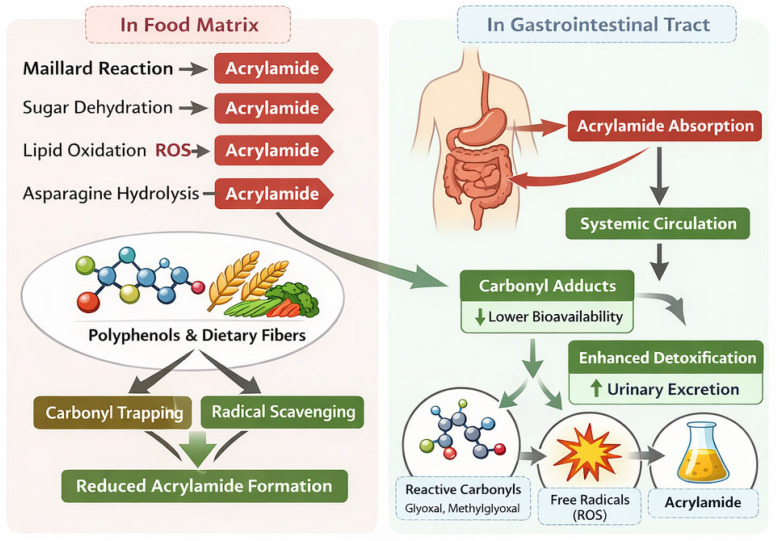
Mechanisms of acrylamide mitigation by dietary antioxidants and polyphenols.

The schematic in Figure 4 illustrates how dietary antioxidants and polyphenols reduce acrylamide formation and bioavailability. In the food matrix, polyphenols and dietary fibers inhibit acrylamide generation via carbonyl trapping and radical scavenging, targeting reactive intermediates from Maillard reactions, sugar dehydration, lipid oxidation, and asparagine hydrolysis. In the gastrointestinal tract, polyphenols further decrease acrylamide bioavailability by forming carbonyl adducts and promoting detoxification through urinary excretion. Key molecular targets involved in these mitigation pathways are highlighted.

### 8.3. Processing Innovations

Recent technological developments in food processing have introduced several innovative strategies to reduce acrylamide content, especially in products subjected to heat treatments. With recent developments in analytical tools such as LC-MS/MS, ELISA, and PCR, highly sensitive detection is possible, even at levels as low as 10 µg/kg, which is imperative in ensuring food safety compliance [95,96,97]. These tools not only improve monitoring but also support validation of mitigation techniques during production. Another major development involves the use of predictive modeling, whereby it is possible for manufacturers to simulate acrylamide formation under different processing scenarios. These models can integrate variables such as temperature, time, moisture levels, and ingredient interactions in predicting levels of acrylamide and optimizing conditions before full-scale production begins [39]. Selective breeding in the production of potatoes with low levels of asparagine is a preventive strategy since it minimizes the levels of acrylamide precursors. In addition, this may be complemented by ingredient modeling and a mechanical check on quality before frying or baking, thus enhancing product safety while maintaining efficiency within diverse food categories [33]. Application of consistent pre-processing standards and use of ingredient modeling allows producers to upscale these mitigation strategies across a wide range of foods [23]. Besides the aforementioned, the application of standardized preprocessing techniques—such as controlled drying, blanching, or soaking—is also scalable and effective. For example, reducing the frying or baking temperature and time in potato and cereal-based products has resulted in up to 70% reductions in the levels of acrylamide [98]. Similarly, soaking raw potatoes prior to frying decreases sugars, leading to a 20–50% reduction, depending on soaking duration and temperature [98]. Taken together, these innovations illustrate how biological, mechanical, and process-driven strategies can be used together to effectively mitigate acrylamide formation throughout the production cycle.

## 9. Consumer Awareness and Education

Despite advancements in detection, significant gaps remain in understanding of how to control and mitigate acrylamide in food, highlighting the need for more exposure data and innovative processing solutions. Raising consumer awareness and promoting cross-sector collaboration are essential to reducing dietary acrylamide through informed choices and safer cooking practices [28].

Effective communication of acrylamide risks requires both transparent labeling and coordinated public education efforts. Clear and standardized food labels can inform consumers about efforts to monitor and reduce acrylamide levels, increasing industry accountability and consumer trust [99,100]. At the same time, comprehensive public health campaigns should raise awareness of acrylamide risks from everyday foods and promote safer cooking practices through collaboration among the government, industry, and academia [101]. Together, these approaches form a dual strategy that strengthens knowledge, transparency, and behavioral change, key steps to reducing acrylamide exposure and achieving safer food for all.

As illustrated in Table 8, labeling and public education serve as parallel pathways that connect research outcomes to consumer behavior, reinforcing the overall food safety framework.

Achieving safer food requires a multidimensional approach built on three interconnected pillars: advancing research and innovation, strengthening cross-sector collaboration, and enhancing consumer awareness and education. Together, these elements form a continuous cycle that supports effective mitigation of acrylamide formation and promotes public health.

Rather than presenting isolated findings, the literature reveals evolving tensions between scientific caution, measurable exposure, and the feasibility of intervention, both for consumers and for producers.

Mucci et al. (2003) [102] provided one of the earliest population-based evaluations following the identification of acrylamide in foods. Their findings suggested no significant association between average dietary intake and cancers of the large bowel, bladder, or kidney. They even observed a negative correlation in some cases, such as in large bowel cancer. Despite recognizing the IARC classification of acrylamide as a probable human carcinogen, the authors highlighted that such classification was grounded in animal models and limited human data, setting a precedent for cautious but non-alarmist communication about dietary risks Subsequent studies challenged this early reassurance by shifting the focus from epidemiological outcomes to ubiquitous exposure. 

In particular, Capez et al. (2015) [103] reported that 91% of Italian breakfast cereals and biscuits tested positive for acrylamide, with levels varying considerably based on ingredients and processing. Their work emphasized that even lower concentrations, if consumed frequently, could contribute to long-term cumulative risk, and underscored the need for reformulation efforts and public education, especially regarding children’s dietary habits. 

Expanding on this, Lee and Kim (2020) [104]. introduced a demographic dimension to the exposure narrative. Their findings in Korea showed that toddlers had the highest intake per body weight, largely due to products such as biscuits and crisps, while coffee was identified as the major source among adults. Their analysis reinforced prior concerns about cereal-based products but further highlighted the importance of age-specific risk communication and culturally appropriate dietary guidance.

Whereas these studies identified risk patterns, Gunduz (2023) [105] shifted toward practical mitigation, arguing that no one-size-fits-all strategy can eliminate acrylamide across all products. Instead, the author proposed a flexible, product-specific toolbox that includes longer fermentation, reduced baking temperatures, and asparaginase treatment, while acknowledging constraints such as cost, nutritional value, and sensory acceptance. This contribution directly addressed earlier calls for reformulation by assessing what is realistically achievable in commercial production and by closing the gap between risk recognition and action.

Complementing this production focus, Díaz-Ávila et al. (2024) [106] synthesized findings on visual indicators and analytical detection, particularly in deep-fried starchy foods. They confirmed that darker coloration and thicker crusts correlate with higher acrylamide levels, but noted that effective monitoring requires costly, sophisticated instrumentation often unavailable in lower-resource settings. Their review also pointed to global regulatory disparities as barriers to universal mitigation, emphasizing the need for accessible, evidence-based tools and guidance that align with both scientific rigor and regional capacity.

Together, these studies trace a trajectory from uncertain health risk toward measurable exposure, and from general recommendations toward context-sensitive interventions. While perspectives differ in focus, ranging from epidemiology to ingredient-level mitigation, all converge on the need to reduce acrylamide exposure through informed consumer behavior and practical processing adjustments.

Overall, the central challenge remains: to balance current scientific limitations with the urgency of translating available knowledge into meaningful dietary and industrial action.

## 10. General Discussion and Future Research Directions

Over the past two decades, research into acrylamide in food has evolved from the initial discovery of its formation during everyday cooking to an increasingly nuanced exploration of its health implications, analytical detection, and mitigation strategies. The articles reviewed reflect this progression, charting a path from early identification of acrylamide’s culinary origins to contemporary assessments of exposure, regulatory action, and technical control measures, all while continuously refining scientific methods.

Across the reviewed literature, a persistent public health concern has emerged regarding the formation of acrylamide during thermal food processing, with emphasis consistently placed on advancing scientific understanding and regulatory adaptability. A coherent research trajectory is delineated, spanning mechanistic modeling, detection and quantification methodologies, mitigation strategies, and health risk assessments—although the focal points and proposed solutions vary across studies.

A predictive, model-driven understanding of acrylamide formation has been strongly advocated by Balagiannis et al. (2019) [107]., who emphasized kinetic modeling and the identification of critical variables, such as precursor concentration, moisture, and thermal processing conditions. Within this framework, regulatory policies are viewed as needing continuous evolution in response to new mechanistic insights. However, this approach, while analytically rigorous, appears more narrowly scoped when compared to broader frameworks proposed in other studies. 

For instance, Jozinović et al. (2019) [108] also examine formation mechanisms—particularly the Maillard reaction—but extend the discussion to practical mitigation through asparaginase application, alongside consideration of bioavailability, analytical precision, and health impact assessments. While Balagiannis et al. [107] emphasize modeling as the cornerstone of risk assessment, Jozinović et al. [108] call for more multidimensional evidence to inform policy, illustrating a divergence in prioritization between predictive accuracy and applied versatility.

Kaur and Halford (2023) [28] take a different angle, embedding mitigation directly within product development, particularly for wheat-based food products. Here, mitigation strategies are assessed not only for their chemical efficacy but also within broader regulatory and public health frameworks, thereby integrating chemistry, food technology, and policy considerations. In contrast to the mechanism-heavy focus of Balagiannis et al., Kaur and Halford advocate for product-specific interventions tailored to real-world constraints and market feasibility. 

Empirical grounding is provided by Perestrelo et al. (2024) [93], who shift attention to dietary exposure patterns within a national consumption context. Their analysis reveals actual acrylamide levels in commonly consumed foods and demonstrates how culinary practices and nutritional habits modulate the risk. This study underscores the importance of surveillance data in setting realistic regulatory thresholds—a dimension that, while implicit in other works, is here explicitly prioritized.

Overall, while a consensus exists on the urgency of reducing acrylamide exposure, these studies diverge in the levels of intervention (mechanistic, product-level, regulatory, or epidemiological) they prioritize. A productive synthesis may thus lie in integrating these diverse approaches, in which real-world data and mitigation strategies inform modeling efforts, shaped by both scientific insight and consumer-level feasibility. A consolidated view of the current literature highlights key research domains, associated challenges, and strategic directions for future investigation and policy alignment, as summarized in Table 9.

Díaz-Ávila et al. (2024) [106] evaluated accurate, cost-intensive, and product-specific analytical methods such as LC-MS and GC-MS. It can be said that low-resource settings are affected by the absence of standardized global protocols. EU Regulation 2017/2158 provides targeted measures for high-risk products. Further studies are necessary to develop technique-specific strategies and improve the integration of monitoring tools into industrial workflows. It should be mentioned that the integration of nanotechnology into acrylamide risk mitigation can help in the improvement of smart food safety systems. Future studies should focus on the development of safe, biodegradable, and food-grade nanomaterials.

Obtaining information on how acrylamide is absorbed, metabolized, and eliminated from the body, especially its conversion into glycidamide through CYP2E1-mediated oxidation, would provide a clearer and more complete picture of its toxicological effects. Glycidamide is more chemically reactive and genotoxic than acrylamide, as it can form DNA adducts that contribute to mutations and reproductive toxicity. Understanding these processes as they occur in the body is critical for accurately evaluating risks to human health, since they influence actual internal exposure levels and the likelihood of long-term adverse effects [116,117].

Food surveillance data from between 2019 and 2025 indicated a progressive but significant reduction in acrylamide concentrations in both potato- and cereal-based food products, largely attributed to the implementation of EU Regulation 2017/2158 [86]. It introduced benchmark levels and motivated the general adoption of mitigation techniques by industry, with wide-ranging modifications to production protocols. On the other hand, there have been constant or slight increases in acrylamide levels in the cases of coffee and cocoa products, probably reflecting consumer demand for stronger flavors and darker roasting, conditions that need higher processing temperatures.

The meta-analysis of 58 publications that appeared during this period revealed a 15–20% average reduction in acrylamide for the regulated food categories. However, regional differences still exist and indicate the need for greater homogeneity in monitoring systems and policy adaptations at the local level, thus allowing policy implementation and effectiveness to be experienced uniformly across Europe and beyond [118].

A mechanistic underpinning for these quantitative conclusions is provided by the review article “Acrylamide in starchy foods subjected to deep-frying, 20 years after its discovery (2002–2022)”. It is worth noting that crust development, water loss, and surface protein denaturation, along with process time and temperature, were driving factors for acrylamide formation. These factors, if not appropriately controlled, accelerate browning and acrylamide build-up in fried starchy foods in particular [106].

Overall, the more recent literature supports the view that, although regulatory measures and technological developments have contributed importantly to reducing acrylamide levels, further efforts in real-time monitoring, adaptive cooking strategies, and consumer education are still needed for sustained improvement.

## 11. Conclusions

Acrylamide remains a critical foodborne contaminant with well-documented carcinogenic, neurotoxic, and reproductive risks, arising primarily from thermal processing of carbohydrate-rich foods. Over the past two decades, advances in analytical detection (LC–MS/MS, ELISA) and mechanistic modeling have substantially deepened understanding of its formation dynamics, particularly through Maillard-driven asparagine–sugar interactions. Nevertheless, key uncertainties persist concerning bioavailability, metabolic conversion to glycidamide, and internal dose variability among populations and food matrices. Recent evidence demonstrates that dietary polyphenols and fibers can effectively mitigate acrylamide absorption and promote metabolic detoxification via carbonyl trapping, radical scavenging, and enhanced urinary excretion of conjugated metabolites, reducing exposure by up to 60% in controlled studies. Yet, despite technological and biological progress, standardized monitoring systems remain inconsistent across food categories and regions, limiting accurate exposure assessment and international comparability.

Achieving meaningful risk reduction will therefore depend on a multidisciplinary integration of strategies, combining predictive kinetic modeling, green processing technologies (such as vacuum frying, ohmic heating, and pulsed electric fields), and real-time industrial monitoring within the framework of regulatory harmonization. Equally crucial is sustained consumer education and transparent labeling, enabling informed choices that complement industrial and policy interventions. Continued collaboration across research, regulation, and production will be essential to minimizing dietary acrylamide exposure and safeguarding long-term public health.

## Figures and Tables

**Table 2:** Representative acrylamide concentrations in major food groups reported in the literature.

Food Group	Typical Acrylamide Range (µg/kg)	Exposure Category	Key Influencing Factors	Ref.
Vegetable chips	<750	High	Asparagine and sugar content of the vegetables, frying conditions, lack of low-acrylamide cultivars, and consumer consumption patterns.	[43]
Breakfast cereals	<200	High	Type of cereal (kamut, spelt, wheat), formulation composition, moisture content, and thermal processing conditions such as baking temperature and time.	[44]
Biscuits and cookies	<20 to 2144 µg/kg	Moderate to high	Dough composition, type of cereal or pseudo-cereal (e.g., rye, teff, oat), thermal treatment during baking, low moisture content, and consumption rate.	[45]
French fries	<20–1068	Low to high	The reducing sugar content of the raw potato, moisture content, the color parameter of the fries, frying conditions, and operational procedures in food service establishments.	[46]
Potato crisps and snacks	21 to 3444	Moderate to high	Type of snack (potato crisps > other snacks), processing method (frying/baking), brand variability, and age-dependent consumption patterns.	[47]
Roasted coffee (dry)	500 to 3800	Moderate to high	Roasting time and temperature, coffee species (Robusta vs. Arabica), and precursor levels in raw beans (especially asparagine and sugars).	[48]
Instant coffee (dry)	<200	High	Roasting intensity, brand differences, and coffee bean origin.	[49]
Cocoa and chocolate products	<30 to 490	Low to high	Higher acrylamide in semi-finished cocoa suggests roasting, conching, and formulation drive its formation.	[50]

**Table 3:** Summary of animal carcinogenicity studies.

Study Type	Species	Exposure Route	Tumor Site	Dose Range (mg/kg/day)	Ref.
Carcinogenicity	*Rattus norvegicus*	Toxicity in the liver	Necrosis, granular cytoplasmic changes, and vacuolar degeneration in liver tissue	2.0 mg/kg/day	[55]
Neurotoxicity	Male SD rats	Chronic acrylamide exposure	Dopaminergic neuron loss, neuroinflammation, and motor impairment	0 mg/kg/day 0.5 mg/kg/day 5 mg/kg/day	[56]
Carcinogenicity	Wistar Han male and female rats	In utero exposure	Fibroadenomas of the mammary gland and thyroid gland follicular tumors	0.5 mg/kg/day 1.5 mg/kg/day 3 mg/kg/day	[57]
Chronic toxicity and oncogenicity study	Fischer 344 rats	Chronic exposure	Tunica vaginalis mesothelioma	0.01 mg/kg/day 0.1 mg/kg/day 0.5 mg/kg/day 2.0 mg/kg/day	[57,58,59]
Carcinogenicity and mutagenicity	Adult and neonatal mice	Synthesis and spectroscopic characterization of DNA adducts from reaction of GA with individual deoxynucleosides	Genotoxic, formation of N7-(2-carbamoyl-2-hydroxyethyl) guanine (N7-GA-Gua) in vivo	0 mg/kg/day 1 mg/kg/day 10 mg/kg/day 50 mg/kg/day	[60]
Gene expression, neurochemistry, hormones, and histopathology	Male Fischer 344 rats	Subchronic acrylamide exposure	Cancer through endocrine disruption	2.5 mg/kg/day 10 mg/kg/day 50 mg/kg/day	[61]
Carcinogenicity	Sprague Dawley rats	Endocrine exposure	Thyroid, adrenal glands, and testis	5 mg/kg/day 10 mg/kg/day 15 mg/kg/day	[62]

**Table 4:** Key findings from neurotoxicity studies.

Model System	Acrylamide Concentration and Exposure Duration	Observed Effect	Proposed Mechanism	Ref.
PC12 cells	0.6 mM 1.25 mM 2.5 mM 5 mM for 24 h	Oxidative stress	ROS increased, MDA increased, GSH decreased; HO-1, NQO-1 increased; NF-kB (IkBa, p65), ERK1/2, JNK, and p38 increased. The MAPK pathway is a regulator of the upstream NF-κB and Nrf2 pathways.	[66,67]
SH-SY5Y human neuroblastoma cells/U251	0–500 μg/mL for 1, 3, or 5 days	Excitotoxicity and neuronal damage	EAAT2 dysfunction (decrease in EAAT2 expression).	[68]
PC12 cells	0.5 mM for 12 h	Apoptosis	Phosphorylation of MAPKs significantly increases.	[69]
BV-2 cells	0.5 mM 1 mM 2 mM for 24 h	Apoptosis	BDNF, Bcl-2/Bax, and p-Akt/Akt decreased. Cyto-c, cleavage-caspase-9, cleavage-caspase-3, and PARP increased. Mitochondrial respiration and anaerobic glycolysis decreased.	[66,70]
H1 hESC cells	2.5 mM 5 mM for 24 h	Apoptosis and Oxidative stress	SOX2, TUJ1, GFAP, CTIP2, and SOX9 decreased. MAPK and Nrf2 increased. FTL, GCLC, GCLM, SLC7A11, and HMOX1 increased. Caspase-6, caspase-9, and c-FOS increased. Stimulated Tau hyperphosphorylation and suppressed neuronal differentiation.	[66,71]
Gastrocnemius motor plate in rats	9 mg/kg 18 mg/kg 36 mg/kg for 21 days	Toxic to the motor plate	Changes in the structure of muscle fibers and nerve endings, resulting in AChE content.	[72]
Male C57BL/6 mice	20 mg/kg/day for 4 weeks	Autophagy	ATG4B, LC3-II, Cathepsin D, and LAMP2a increased. Trx-1 siRNA enhances ACR-induced autophagy by regulating ITGAV.	[66]
Male SD rats	50 mg/kg/day for 3–28 days	Neurotransmitter dysfunction	Neurological toxicity and weight loss. ACR-cysteine adduct (CEC) and 7S SNARE core complex increased.	[64,66]
Male C57/BL6J mice	Drinking water containing 0.003% acrylamide for 16 weeks	Brain–gut axis inflammation	Bmal1, Clock, SNAP-25, PSD-95, ZO-1, and Occludin decreased. IL-10, COX-2, TNF-a, and COX-2 increased.	[66,73]
Dosage-dependent neurotoxicity in humans	Daily high-dosage exposure	Numbness in limbs, muscular weakness, cognitive impairment, and axonal neuropathy	Terminal nerve damage in the PNS and CNS.	[66]
Human embryonic stem cells (H1hESC)	2.5 mM and 5 mM for 24 h	Oxidative stress response	ACR inhibited neuron differentiation.	[66,71]
Barber and LoPachin exposed Sprague Dawley (SD) rats	50 mg/kg/day for 28 days	ACR neurotoxicity	Weight loss and abnormal gait.	[64]
Adult male SD rats	40 mg/kg/day ACR for 4 weeks	Oxidative stress	Death of hippocampal neurons and neurotoxicity.	[66]

**Table 5:** International benchmarks for acrylamide in foods.

Region/ Authority	Product Category	Benchmark Level (µg/kg)	Region/ Authority	Legal Status/ Acrylamide Content in Food	Ref.
United Union	Potable water	Maximum allowable concentration is 0.1 µg/dm^3^	Environmental Protection Agency (EPA)	Guidelines set the amount at 0.5 µg/dm^3^	[72]
Regulation (EU) 2017/2158	Wheat-based bread	50 μg/kg	European Union	70–430 μg/kg	[72,79,80,81]
	Breakfast cereals	300 μg/kg	European Union	30–1400 μg/kg	
	Biscuits and cookies	350 μg/kg	European Union	<30–3200 μg/kg	
	Fried potato products (except potato crisps and snacks)	500 μg/kg	European Union	200–2287 μg/kg	
	Potato crisps and snacks	750 μg/kg	European Union	<50–3500 μg/kg	
	Roast coffee (dry)	400 μg/kg	China	16.5–263 μg/kg	[80,81,82]
	Instant coffee (dry)	850 μg/kg	China	32.2–673 μg/kg	

**Table 6:** Overview of international and national acrylamide risk management approaches.

Organization	Approach	Strategies	Notes	Ref.
Codex Alimentarius Commission (CAC)	Voluntary Code of Practice	- Good manufacturing practices (GMP) - Process control - Raw material quality	Supports global harmonization and fair trade; non-binding guidance (Codex Alimentarius Commission, 2009)	[83]
U.S. Food and Drug Administration (FDA)	Non-binding guidance	- Encourages voluntary mitigation - Continuous reduction in AA levels - Focus on general and vulnerable populations	No maximum limits; emphasizes shared responsibility (U.S. FDA, 2016)	[84]
JECFA (FAO/WHO)	Scientific risk assessment	- Classification as a genotoxic carcinogen - Margin of Exposure (MOE) approach - ALARA principle	Provides toxicological basis for global regulatory decisions (JECFA, 2013)	[85]
European Commission (EU)	Binding Regulation (EU 2017/2158)	- Implementation of mitigation strategies - Monitoring of AA levels in foods	Benchmark values for potato products, bakery items, cereals, coffee, baby foods; reference values, not legal limits	[19]

**Table 7:** Summary of key studies on cooking techniques influencing acrylamide formation in fried starchy foods.

Year	Study	Specific Findings	Ref.
2013	In-house–validated LC-MS/MS method for survey of acrylamide in various processed foods from the Korean market	Acrylamide (AA) was detected in 274 Korean processed food samples at levels from below the detection limit to 1435 µg/kg, with the highest concentrations found in potato chips and French fries, followed by biscuits and tea, and LC-MS/MS analysis showed excellent repeatability (RSD < 5%) and recoveries of 94.5–107.6%, enabling sensitive quantification down to 10 µg/kg.	[90]
2018	Effect of Microwave Frying on Acrylamide Generation, Mass Transfer, Color, and Texture in French Fries	Microwave frying of potato strips at 315–600 W reduced acrylamide by 37–83% compared to deep-oil frying, producing potatoes with moisture and texture similar to chips, with a fat content below 20 g/100 g and acrylamide levels under 100 µg/kg.	[91]
2023	Comparative study of conventional frying and air frying on the quality of potatoes	Air-fried French fries contained about 48% less moisture than conventionally fried fries, showed fewer color changes, less surface damage, improved crunchiness, and more stable thermal and chemical properties, highlighting air frying as a viable alternative to reduce oil-related quality changes.	[92]
2024	Results of the BfR MEAL Study: Acrylamide in foods from the German market, with the highest levels in vegetable crisps	In 230 foods analyzed in the German Total Diet Study, acrylamide levels were highest in vegetable crisps (1430 µg/kg), followed by potato pancakes (558 µg/kg) and pan-fried potatoes (450 µg/kg), with French fries exceeding EU benchmark levels at browning degree 3, while oven-baked fries and air-fried sweet potatoes had the lowest levels, and popcorn, salty sticks, and dark chocolate contained 243, 190, and 130 µg/kg, respectively.	[93]

**Table 8:** Integrated communication strategies leading to safer food.

Labeling Practices	Public Health Campaigns
Indicate monitoring and reduction efforts	Educate consumers about acrylamide risks and safe cooking practices
Provide clear and consistent definitions	Foster joint efforts among health authorities and industry
Use standardized terminology	Promote safer cooking and ingredient choices
Present informative yet concise processing information	Develop uniform safety and awareness practices
Improve transparency and strengthen consumer trust	Encourage industry-wide collaboration and continuous monitoring

**Table 9:** Emerging research priorities and knowledge gaps in acrylamide risk mitigation.

Research Area	Key Focus	Challenges	Future Directions	Ref.
**Formation** **Mechanisms**	Understanding acrylamide behavior in food matrices	Limited knowledge on interactions with lipids and molecular cross-linking	Investigate formation dynamics in complex matrices and lipid-rich environments.	[35]
**Detection and Quantification**	Improving analytical accuracy and QA/QC	Ineffective extraction in high-fat foods; lack of harmonization	Develop robust QA/QC protocols; validate new methods (HPLC, GC–MS) across food types.	[109]
**Green and** **Innovative** **Mitigation**	Reducing acrylamide formation during processing	Traditional methods are energy-intensive	Employ green techniques (vacuum frying, ohmic heating, PEF pretreatments); integrate AI-assisted modeling.	[95]
**Real-Time** **Monitoring**	Online control of food contaminants	Limited real-time surveillance systems	Implement sensor-based, Industry 4.0-integrated monitoring for Maillard contaminants.	[110]
**Epidemiology and Health Risk**	Understanding exposure and carcinogenicity	Inconclusive human data; variability in exposure	Improve exposure assessment using biomarkers (e.g., hemoglobin adducts) and refined food-frequency tools.	[111]
**Related** **Compounds**	Study of co-occurring contaminants	Insufficient research on 5-HMF and similar molecules	Examine 5-HMF roles in carcinogenesis and chronic disease pathways.	[112]
**Industrial** **Application**	Scaling lab findings to production	Lab-scale solutions are complex to apply in industry	Bridge the lab–industry gap; optimize frying conditions, oil blending, and additive use (citric acid, asparaginase).	[113]
**Advanced** **Analytical Tools**	Enhancing detection precision	Need for high-sensitivity methods	Utilize LC–MS/MS to detect acrylamide at ultra-trace levels (≤0.5 µg/kg) for strict QA.	[114]
**Global Collaboration and Regulation**	Coordinating food safety responses	Regional differences in regulation	Strengthen international harmonization (e.g., CODEX, EU initiatives)	[115]

## Data Availability

No new data were created or analyzed in this study. Data sharing is not applicable to this article.
